# Tumor-infiltrating B cells and T cells correlate with postoperative prognosis in triple-negative carcinoma of the breast

**DOI:** 10.1186/s12885-021-08009-x

**Published:** 2021-03-16

**Authors:** Hajime Kuroda, Tsengelmaa Jamiyan, Rin Yamaguchi, Akinari Kakumoto, Akihito Abe, Oi Harada, Atsuko Masunaga

**Affiliations:** 1 Department of Diagnostic Pathology, Tokyo Women’s Medical University, Medical Center East, 2-1-10 Nishiogu, Arakawa-ku, Tokyo, 116-8567 Japan; 2 Department of Diagnostic Pathology, Dokkyo Medical University, Mibu, Japan; 3 Department of Pathology and Forensic Medicine, Mongolian National University of Medical Sciences, Ulan Bator, Mongolia; 4 Department of Pathology & Laboratory Medicine, Kurume University Medical Center, Kurume, Japan; 5 Department of Diagnostic Pathology, Nasu Red Cross Hospital, Otawara, Japan; 6 Breast Center, Dokkyo Medical University, Mibu, Japan; 7 Breast center, Showa University, Tokyo, Japan

**Keywords:** Breast, Triple-negative cancer, Tumor-infiltrating B lymphocytes, Tumor-infiltrating T lymphocytes

## Abstract

**Background:**

In this study, we investigated CD20+ TILs in triple-negative breast cancer (TNBC) and their relationship with T lymphocyte subsets (CD4+, CD8+, CD25+, and FOXP3+), including their combined prognostic value using an immunohistochemical staining method.

**Methods:**

We investigated 107 patients with TNBC for whom a full-face section stained by hematoxylin and eosin between 2006 and 2018 at Dokkyo Medical University Hospital was available.

**Results:**

The strongest association of infiltrating CD20+ TILs was with CD4+ TILs. There was a significant relationship between CD20+ and CD4+ TILs (*r* = 0.177; *p* < 0.001), CD8+ TILs (*r* = 0.085; *p* = 0.002), and FOXP3+ TILs (*r* = 0.0043; *p* = 0.032). No significant relationships were observed between the CD20+ and CD25+ TILs (*r* = 0.012; *p* = 0.264). Multivariate analysis revealed that only the CD20+/FOXP3 ratio was an independent factor for relapse-free survival (*p* < 0.001) and overall survival (*p* < 0.001). Patients with tumors highly infiltrated by CD4+, CD8+, and CD20+ TILs had a good prognosis. In contrast, those with tumors weakly infiltrated by CD20+ TILs but highly infiltrated by CD25+ and FOXP3+ TILs had a poor prognosis.

**Conclusions:**

CD20+ TILs may support an increase in CD4+ and CD8+ TILs, which altered the anti-tumor response, resulting in a positive prognosis. CD20+ TILs correlated with FOXP3+ Treg lymphocytes, which were reported to be correlated with a poor prognosis. Our study suggested that TIL-B cells have dual and conflicting roles in TIL-T immune reactions in TNBC.

## Background

Tumor-infiltrating lymphocytes (TILs) are a major factor in the tumor microenvironment, which is important in the development and progression of cancer. A correlation with prognosis in patients with breast cancer highly infiltrated by TILs was previously reported [1–3]. Furthermore, several immunohistochemical studies concluded that tumor – infiltrating T lymphocytes (TILs-T) have antitumor activity in mammary glands. Most TILs in cancer are T lymphocytes, and include CD4+ (helper) and CD8+ (cytotoxic) lymphocytes [4–9]. CD4+ TILs are necessary to activate proliferation and memory in tumor-specific CD8+ TILs [10]. We previously found that CD4+ and CD8+ TILs are correlated with a favorable prognosis in triple-negative breast cancer (TNBC) [11]. Although the prognostic correlation of TILs-T has been widely reported, there are few studies on infiltrating B lymphocytes (TILs-B) in breast cancer and there is no consensus on their prognostic impact [12–20]. In addition, few studies have investigated TILs-B and TILs-T in combination [21–24]. Indeed, TILs that contain both TIL-B and TIL-T are correlated with lymphocyte proliferation and a good prognosis, suggesting that TIL-B cooperate with TIL-T in an anti-tumor reaction [25]. Among T lymphocytes, T regulatory (Treg) lymphocytes suppress effector T lymphocytes and control immune responses [26]. The mechanisms governing Treg lymphocyte proliferation and function recently attracted attention because of their importance in suppressing the expansion of autoimmunity and their therapeutic viability. In animal models, immune-related tumor clearance and heightened response to immunotherapy were improved by the removal of Treg lymphocytes [27]. Furthermore, high infiltration of Treg lymphocytes was reported to be correlated with a negative prognosis in human cancers, including breast cancer, and they may represent a new therapeutic target [26–31]. Forkhead box protein 3 (FOXP3) and CD25, which are considered the most reliable markers of Treg lymphocytes play an important role in immune-suppression, thereby inducing immune tolerance. However, TIL-B and Treg lymphocyte involvement in human cancer remains unclear.

The correlation between CD20+ TILs and TNBC has not been clarified, and their relationship with other immune cell subsets (CD4+, CD8+, CD25+, FOXP3+) in TNBC has not been reported. This study was carried out to evaluate the behavior of CD20+ TILs in TNBC and their relationship with T lymphocyte subsets (CD4+, CD8+, CD25+, FOXP3+), assessing their prognostic value using an immunohistochemical staining method.

## Materials and methods

### Patients

We investigated 107 patients with TNBC for whom a full-face section stained by hematoxylin and eosin (H&E) between 2006 and 2018 at Dokkyo Medical University Hospital was available. All patients received pre-operative chemotherapy. The clinicopathological variables, including age at the time of diagnosis, tumor size, histology, tumor grade, Mib-1 index, and TILs status, were reviewed.

### Immunohistochemistry (IHC)

IHC was performed using monoclonal antibodies against estrogen receptor (ER) (clone SP1, Novocastra (Leica), prediluted, nuclear), progesterone receptor (PgR) (clone 1E2, Novocastra (Leica), prediluted, nuclear), human epidermal growth factor receptor 2 (HER2) (clone 4B5, Roche (Ventana), prediluted, membranous), CD4 (CD4, clone 1F6, Novocastra (Leica), 1:40), CD8 (CD8, clone 4B11, Novocastra (Leica), prediluted), CD20 (CD20, clone L26, Nichirei), CD25 (CD25, clone 4C9 Novocastra (Leica), prediluted), and FOXP3 (FOXP3, clone 236A/E7, Abcam, 1:50). Hematoxylin was used as a counterstain. ER and PgR expression was estimated, and > 1% nuclear-stained tumor cells was considered positive according to the American Society of Clinical Oncology and the College of American Pathologist (ASCO/CAP) guidelines [32]. HER2-negative status was confirmed as a staining score of 0/1+. In cases with an IHC score of 2, fluorescence in situ hybridization (FISH) was used to determine the HER2 status, and it was considered positive when the ratio of HER2 to chromosome enumeration probe 17 (CEP17) was > 2.0 [33]. The mib-1 index was estimated using a semiquantitative visual method and the threshold was set at 20% based on a previous report [34]. Tumor cells were considered positive for mib-1 only when nuclear staining was notable. TILs in H&E-stained sections were assessed according to the International Immuno-Oncology Biomarkers Working Group [35] and the threshold was set at 30% [36]. Stromal TILs were defined as the percentage of immune cells in the tumor stroma and outside the tumor nests. To evaluate CD4+, CD8+, CD20+, CD25+, and FOXP3+ TILs, the number of positive cells was calculated from IHC staining. Each specimen was screened at low magnification (× 100), and the five areas with the greatest number of positively stained cells in the stroma were selected for further analysis. The mean TIL count in these areas in each case was estimated at high (× 400) magnification. Moreover, the ratios of CD20/CD4, CD20/CD8, CD20/CD25, and CD20/FOXP3 were calculated. Statistically, the number of positive cells was classified into low and high groups based on a threshold assessed by the median (Table 1). Each specimen was analyzed by two investigators (TJ and HK) who were blinded to the clinicopathological information, and an average of the results was obtained.

**Table 1:** Distribution pattern of stromal TILs in TNBC

Variables	Mean	SE	Median	Range
CD4+ TILs	105.07	5.56	104	273
CD8+ TILs	91.79	5.02	81	251
CD20+ TILs	79.43	7.19	60	276
CD25+ TILs	21.47	1.49	16	66
FOXP3+ TILs	38.27	2.89	31	153

*TILs* tumor-infiltrating lymphocytes, *TNBC* triple-negative breast cancer, *SE* standard error

### Statistical analysis

The correlation between the number of immune cells (CD4+, CD8+, CD20+, CD25+, and FOXP3+) and the clinicopathological variables was analyzed using the chi square test. The Wilcoxon signed ranks test was performed to compare the B and T cell markers. We explored the association between CD20 and T cell markers using a linear regression model. The Kaplan–Meier method was used to estimate relapse-free survival (RFS) and overall survival (OS), and differences were analyzed by the log-rank test. Univariate and multivariate Cox proportional hazard models were used to assess the hazard ratios (HRs) with a confidence interval (CI) of 95% for survival and *p* < 0.05 was considered significant. We converted continuous variables into categorical variables in the Cox regression model, which was adjusted for relevant clinical covariates, including age, tumor size, histological grade, and lymph node status. Statistical analyses were carried out using IBM SPSS Statistics 26 (IBM, Armonk, NY, United States).

## Results

**Fig. 1:**
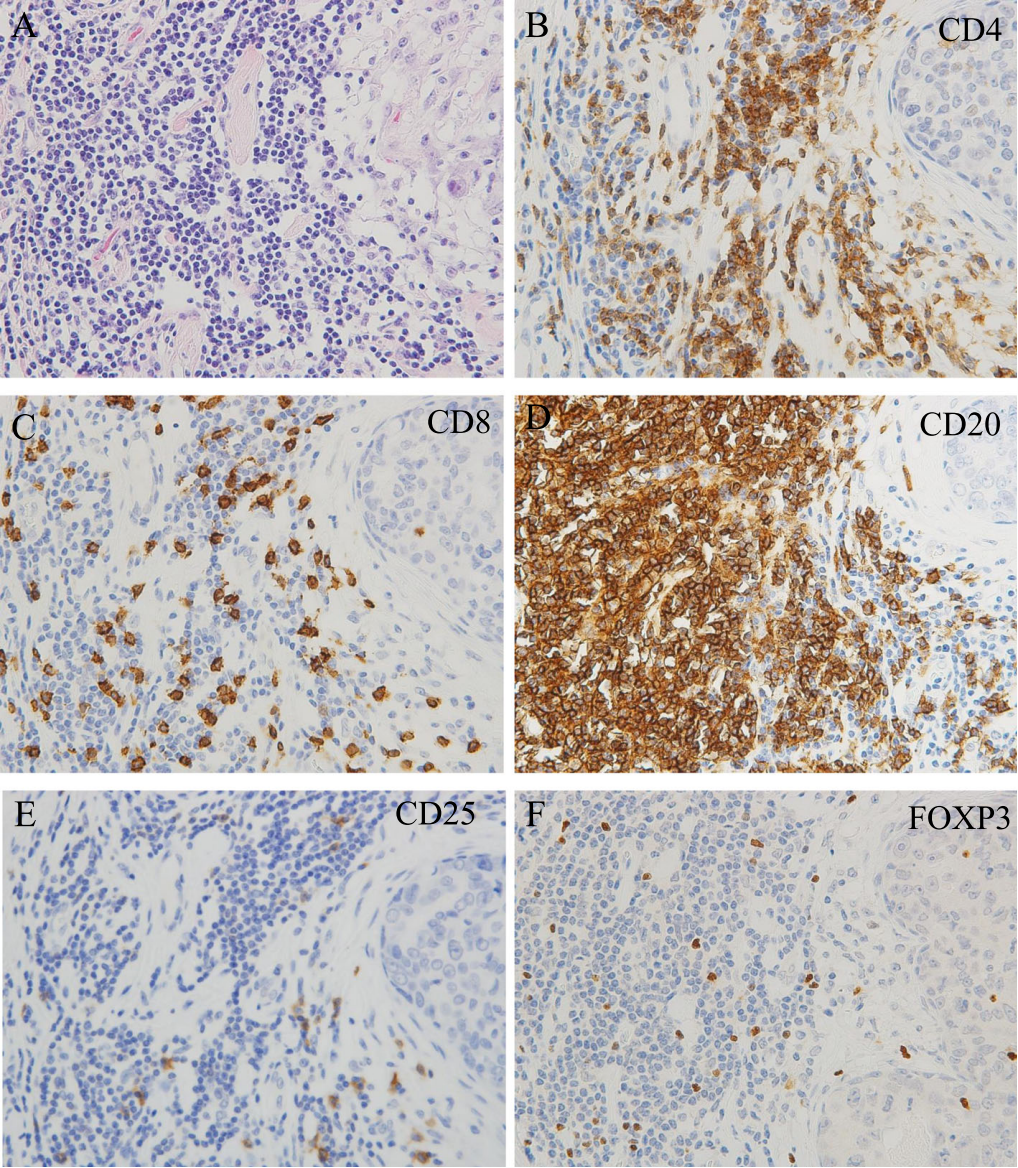
Representative photomicrographs of TILs in patients with TNBC. A patient with a high level of TILs on HE staining (**a**). IHC using primary antibodies against CD4 (**b**), CD8 (**c**), CD20 (**d**), CD25 (**e**), and FOXP3 (**f**) to characterize TILs in the same section of tumor tissue (original magnification, × 400)

**Fig. 2:**
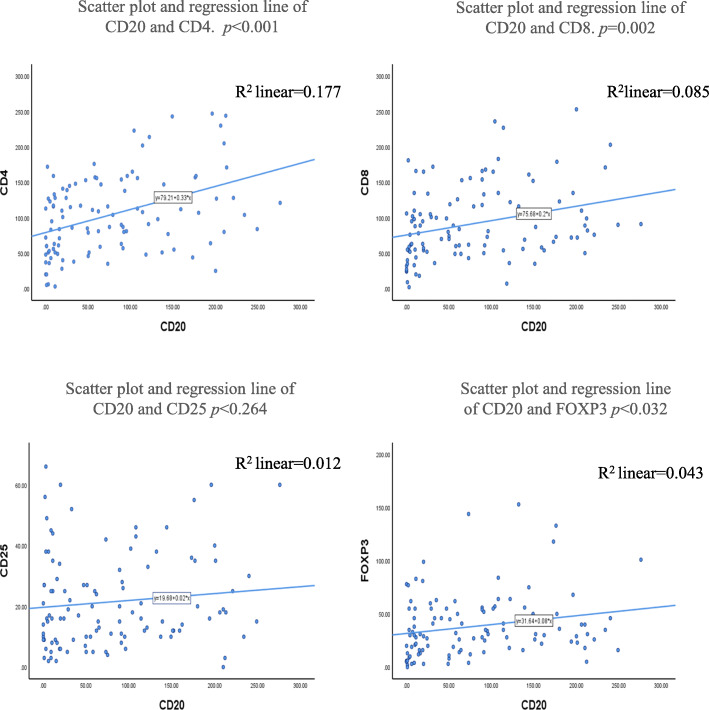
Absolute counts of CD20+ TILs and CD4+, CD8+, CD25, and FOXP3+ TILs in the peripheral stroma of patients with TNBC. The *horizontal* and *vertical reference lines* display absolute numbers of CD20+ TILs and CD4+, CD8+, CD25+, and FOXP3+ TILs. As shown by the *regression lines*, counts of CD4+, CD8+, and FOXP3+ TILs increased along with CD20+ TILs in TNBC. However, the counts of CD25+ were not related to CD20+ TILs

**Fig. 3:**
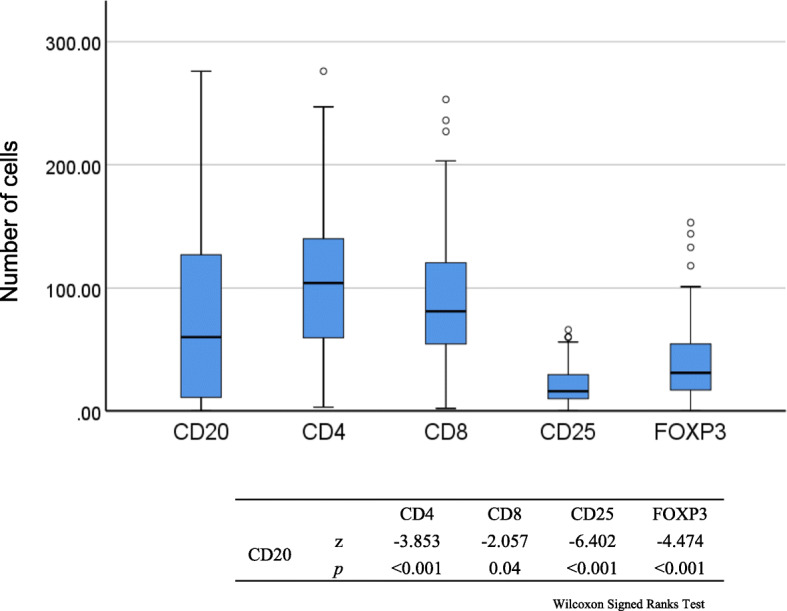
*Box plots* displaying the correlation of CD20+ TILs with the absolute numbers of CD4+, CD8+, FOXP3+, and CD25 TILs in patients with TNBC. The *bars* are median values, the *box* displays the interquartile range (25–75%), and the whiskers extend to 1.5 x the interquartile range

We investigated the populations of infiltrating immune cells in TNBC. Immune cell subsets were distinguished by staining of CD4 (T helper lymphocytes), CD8 (cytotoxic T lymphocytes), CD20 (B lymphocytes), and CD25 or FOXP3 (Treg lymphocytes) (Fig. 1). The strongest association of infiltrating CD20+ TILs was with CD4+ TILs (Fig. 2). There was a significant relationship between CD20+ and CD4+ TILs (r^2^ = 0.177; *p* < 0.001), CD8+ TILs (r^2^ = 0.085; *p* = 0.002), and FOXP3+ TILs (r^2^ = 0.043; *p* = 0.032). However, no significant relationships were observed between the CD20+ and CD25+ TILs (r^2^ = 0.012; *p* = 0.264) (Fig. 2). The correlation with CD20+ TILs of the total number of CD4+, CD8+, CD25+, and FOXP3+ TILs (*p* < 0.001, *p* = 0.04, *p* < 0.001, and *p* < 0.001) in patients with TNBC was examined (Fig. 3). Overall, T helper lymphocytes were the main immune cells infiltrating the TNBC stromal region, and these included cytotoxic T lymphocytes, B lymphocytes, and Treg lymphocytes. Infiltration of CD20+ TILs was separated into low and high groups to adapt to the median threshold, and analyzed for correlations with the clinicopathological features (Tables 1 and 2). As a result, the threshold was 104, 81, 60, 16, and 31 for CD4, CD8, CD20, CD25, and FOXP3, respectively. A high density of CD20+ TILs was significantly related to a high density of TILs (*p* < 0.001). However, there was no significant relationship between a high density of CD20+ TILs and other clinicopathological characteristics (age at the time of diagnosis, tumor size, tumor grade, histology, lymph node metastasis, and mib-1 index).

**Table 2:** Clinicopathological characteristics of TNBC and the status of stromal CD20 (*n* = 107)

Clinicopathological characteristics	Total no. of cases	CD20		
		Low	High	*P* value
Age (years)				0.499
< 60	53	25	28	
≥ 60	54	29	25	
Tumor size (cm)				0.087
≤ 2	66	29	37	
> 2	41	25	16	
Tumor grade				0.564
I & II	31	17	14	
III	76	37	39	
Histology				0.806
IDC	82	40	42	
ILC	2	1	1	
other types	23	13	10	
Lymph node metastasis				0.952
absent	77	39	38	
present	30	15	15	
Mib-1 index				0.475
< 20%	19	11	8	
≥ 20%	88	43	45	
TILs				< 0.001
low	58	44	14	
high	49	10	39	

*TNBC* triple-negative breast cancer, *IDC* invasive ductal carcinoma, *ILC* invasive lobular carcinoma, *TILs* Tumor-infiltrating lymphocytes

χ2 test

**Fig. 4:**
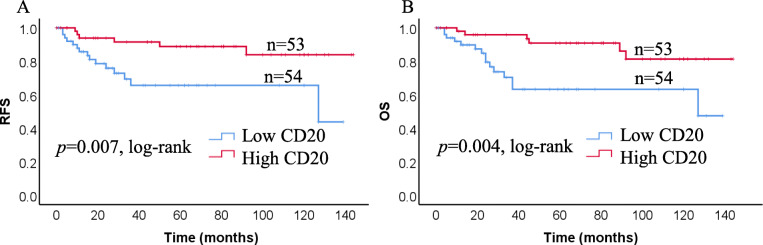
Kaplan-Meier plots showing RFS and OS in TNBC according to infiltration of CD20+ TILs. Log-rank tests were used to estimate *P*-values

Next, we analyzed the correlation of infiltrating CD20+ TILs with RFS (*p* = 0.007) and OS (*p* = 0.004) in patients with TNBC. Patients with tumors highly infiltrated by CD20+ TILs had a good prognosis (Fig. 4). We analyzed the prognostic value of CD20 + TILs compared with other investigated immune cell subsets (Table 3). In univariate survival analyses, the CD20+/CD4+ and CD20+/CD8+ ratios were not significantly associated with RFS (*p* = 0.348, and *p* = 0.319) or OS (*p* = 0.406 and *p* = 0.274). However, lower CD20+/CD25+ and/or CD20+/FOXP3 ratios were correlated with a poorer RFS (*p* = 0.009, and *p* = 0.001) and OS (*p* = 0.007 and *p* = 0.001). By multivariate analysis, only a lower CD20+/FOXP3+ ratio was an independent factor for a poorer RFS (*p* < 0.001) and OS (*p* < 0.001).

**Table 3:** Cox regression analyses of infiltrating immune cells in RFS and OS of TNBC patients

Immune cell markers	Univariate analysis						Multivariate analysis					
	RFS			OS			RFS			OS		
	HR	95% CI	*P* value	HR	95% CI	*P* value	HR	95% CI	*P* value	HR	95% CI	*P* value
CD20/CD4	1.618	0.592–4.419	0.348	1.532	0.560–4.190	0.406	2.797	0.855–9.151	0.089	1.898	0.557–6.467	0.306
CD20/CD8	0.643	0.270–1.532	0.319	0.615	0.258–1.468	0.274	2.915	0.941–9.031	0.064	3.147	0.936–10.57	0.064
CD20/CD25	0.261	0.095–0.717	0.009	0.247	0.090–0.678	0.007	1.082	0.303–3.857	0.903	0.694	0.191–2.527	0.58
CD20/FOXP3	0.085	0.020–0.367	0.001	0.083	0.019–0.361	0.001	0.028	0.005–0.166	< 0.001	0.044	0.008–0.246	< 0.001

*RFS* relapse-free survival, *OS* overall survival, *TNBC* triple-negative breast cancer, *HR* hazard ratio, *CI* confidence interval

**Fig. 5:**
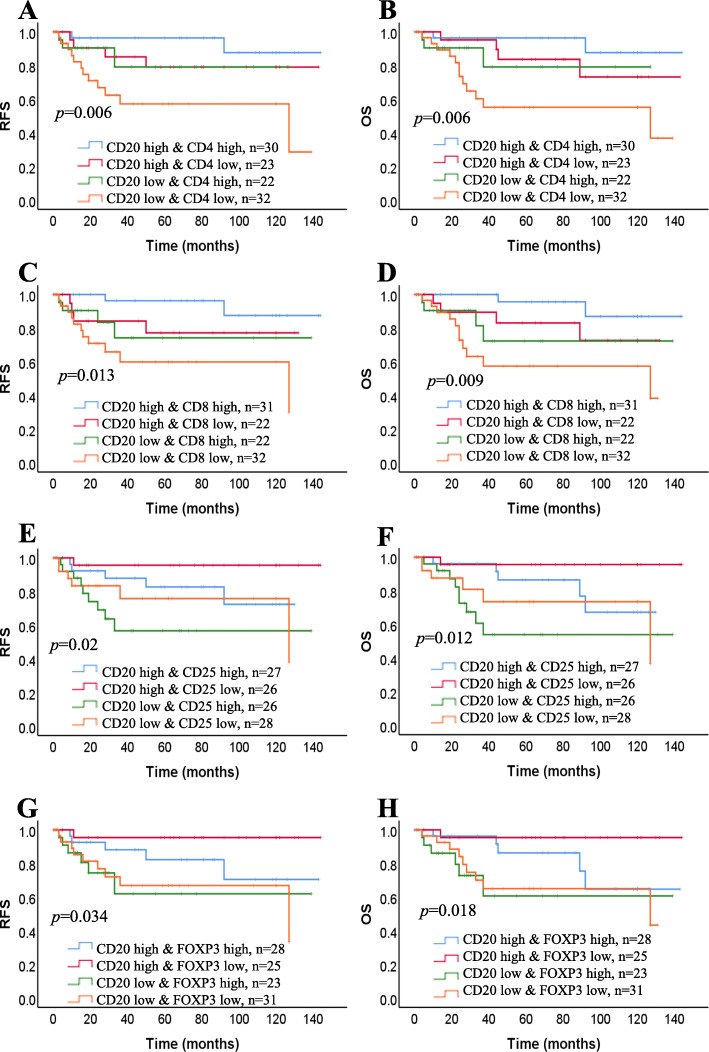
Densities of TIL-B and TIL-T are associated with patient survival. Kaplan-Meier curves showing RFS and OS of the patients by combining CD20+ with CD4+, CD8+, CD25+, and FOXP3+ TILs. **a**, **b** CD20-high CD4-high (*n* = 30), CD20-high CD4-low (*n* = 23), CD20-low CD4-high (*n* = 22), CD20-low CD4-low (*n* = 32). **c**, **d** CD8-high CD4-high (*n* = 31), CD20-high CD8-low (*n* = 22), CD20-low CD8-high (*n* = 22), CD20-low CD8-low (*n* = 32). **e**, **f** CD20-high CD25-high (*n* = 27), CD20-high CD25-low (*n* = 26), CD20-low CD25-high (*n* = 26), CD20-low CD25-low (*n* = 28). **g**, **h** CD20-high FOXP3-high (*n* = 28), CD20-high FOXP3-low (*n* = 25), CD20-low FOXP3-high (*n* = 23), CD20-low FOXP3-low (*n* = 31). Graph shows p = log-rank test *P*-values

To analyze the prognostic value of TILs-B in correlation with infiltrating TILs-T, we investigated the prognostic impact of groups of CD20+ TILs (high or low) compared with that of CD4+, CD8+, CD25+, and FOXP3+ TILs (high or low). Patients with tumors highly infiltrated by CD4+ and CD20+ TILs had a better prognosis than those with tumors highly infiltrated by CD4+ TILs but weakly infiltrated by CD20+ TILs (Fig. 5a and b). In patients with tumors weakly infiltrated by CD4+ TILs, the prognostic value of CD20+ TILs decreased. Almost identical correlations were noted between CD20+ TIL infiltration and CD8+ TIL infiltration (Fig. 5c and d). Of note, in patients with tumors weakly infiltrated by CD20+ TILs but highly infiltrated by CD25+ and FOXP3+ TILs, the opposite effect was observed (Fig. 5e-h).

## Discussion

In this study, we investigated TILs-B in TNBC and their relationships with T lymphocyte subsets. We examined the prognostic role of groups of CD20 (high and low) combined with those of CD4 (high or low) and CD8 (high or low) in the stromal component using Kaplan-Meier curves. CD20+ TILs were associated with CD4+ and CD8+ TILs. A good prognosis was observed in TNBC in which tumors were highly infiltrated by CD20+ TILs combined with CD4+ and CD8+ TILs; therefore, CD20+ TILs may support CD4+ and CD8+ TILs in altering the anti-tumor response.

Correlations of TILs-B with TILs-T were previously reported in liver, ovary, and colon carcinomas [21–24]. Shi et al. used IHC to examine the population and prognostic significance of CD20+ TILs in a hepatocellular carcinoma series [23]. A high density of TILs-B in the tumor margin induced an increase in stromal CD8+ TILs. Nielsen et al. also reported an association between stromal CD20+ and CD8+ TILs in ovarian carcinoma [21]. Recently, CD20+ TILs were reported to be highly and positively correlated with CD8+ TILs in large colon cancer series [24]. As mentioned above, there are several reports of an association between TIL-B and CD8+ TILs in human diseases, including cancer. However, there are few reports on TILs-B and their influence on CD4+ TILs. Similar to CD8+ TILs, CD20+ TILs were reported to be associated with CD4+ TILs in non-small cell lung cancer [37]. B lymphocytes can support anti-tumor activity through several mechanisms. This concept is supported by studies demonstrating that deficient T cell responses are correlated with B cell deficiency in gene-disrupted mice [38, 39]. B lymphocytes from tumor-bearing or naïve donors played similar roles in suppressing tumor growth when transplanted with T lymphocytes [39]. Furthermore, in B lymphocyte-deficient mice produced by continuous injection of anti B antibodies, T lymphocytes activated in lymph nodes relied on antigen presentation by B lymphocytes. This suggests that B lymphocytes capture antigens and present them to T lymphocytes after transplantation into the recipient mice. B lymphocytes are assumed to bind tumor antigens through surface immunoglobulin (Ig) molecules, process them, and then present tumor-derived antigens through major histocompatibility complex classes I and II to induce the activation of both CD8+ cells and CD4+ T lymphocytes [40].

In vivo, coordinated B and T lymphocyte reactions were previously reported in both auto-immunity and allograft rejection [41, 42]. In allograft rejection and chronic infection, B lymphocytes are observed in the central tertiary lymphoid structures in affected tissues [43]. In a xenograft model of rheumatoid arthritis, the loss of B lymphocytes reduced T lymphocyte activation and proliferation in stroma according to anti-CD20 antibody staining. Furthermore, the loss of T lymphocytes induced the breakdown of tertiary lymphoid structures and depletion of Ig immunoglobulin production by B lymphocytes [44]. These reports support our results that TILs-B are associated with stroma CD4+ and CD8+ TILs in TNBC. TILs-B may induce Treg lymphocyte development and proliferation, and affect the prognosis. In our study, CD20+ TILs were associated with FOXP3+ TILs expression, but not with CD25+ TILs. However, in the multivariate analysis, CD20+ and FOXP3+ TILs had an opposing relationship. Furthermore, CD25+ and FOXP3+ TILs had no prognostic advantage based on Kaplan-Meier curves. High infiltration of Treg lymphocytes was reported to correlate with the prognosis in human cancers [11, 26–31]. We previously reported that TNBC is associated with FOXP3+ TILs [11]. In addition, the distribution of circulating CD4 + CD25 + FOXP3+ Treg lymphocytes and CD19 + CD24 + CD38 + B lymphocytes significantly increased in breast cancer patients compared with healthy controls using blood samples [45]. CD19+ B lymphocytes induced the expansion of Treg lymphocytes expressing FOXP3 in in vitro assays [46]. Furthermore, stimulated B lymphocytes were used to induce Treg lymphocytes [47]. Co-culture of CD40L-activated B lymphocytes resulted in an increased number of CD4+ T lymphocytes that also expressed CD25 and FOXP3. These Treg lymphocytes inhibited the development of T lymphocytes by reacting with the original target alloantigens expressed on the B lymphocytes. This tolerance theory is reasonable based on our study demonstrating that CD20+ TILs are correlated with a positive prognosis and increase the number of FOXP3+ Treg lymphocytes. Therefore, our study suggested that TILs-B have dual and conflicting roles in TILs-T immune reactions in TNBC.

However, the counts of CD25+ TILs, which are also considered to be a Treg lymphocyte marker, were not related to CD20+ TILs. FOXP3 is considered to be the most reliable Treg lymphocyte marker in human cancer [48, 49]. However, it is expressed by Treg lymphocytes, epithelial tumor cells, and primed CD4+ and CD8+ lymphocytes. Therefore, several previous studies included CD25+ as a marker of Treg lymphocytes. Although CD25 is expressed by effector T lymphocytes like FOXP3, it is unclear whether CD25 more accurately estimates Treg lymphocytes. Based on previous reports, the differences in Treg lymphocytes may be due to the marker used.

There are some limitations to this study. We found only a weak-fair correlation by scatter plot and the regression line among CD4, CD8, CD25, FOXP3, and CD20 expression. A possible explanation is the small number of TNBC cases examined in this study. The low r^2^ graphs show that even noisy, high - variability data can have a significant tendency. However, the tendency indicates that CD20 still influences CD4, CD8, and FOXP3. Therefore, the outcome would likely be clearer if a greater number of TNBCs were examined.

Another limitation is that we used a visual assessment of TILs. However, there is no standard visual assessment estimation method for TILs as compared with a digital assessment system. At present, the visual assessment continues to have inherent limitations that cannot be fully addressed through standardization and training. In recent years, some researchers have estimated TILs by digital assessment systems. However, they also have some limitations. The International Immuno-Oncology Biomarker Working Group recommended that digital pathology algorithms need to account for the complexity involved in TIL-scoring procedures by a pathologist [50]. However, even validated stand-alone digital assessment requires checks by pathologists to prevent unexpected failures. Further, a Wouters et al. meta-analysis article reported scoring of CD20+ TIL either visually or digitally using various software packages and found no differences in the direction of the outcome data for either of these methods [51]. Therefore, it is unavoidable that there is some ambiguity with all methods. Currently, pathologists need to accept that there is some ambiguity in terms of estimation.

## Conclusion

Our study provides evidence of a correlation between TILs-B and TILs-T in TNBC patients. CD20+ TILs were associated with CD4+, CD8+ and FOXP3+ TILs, but not with CD25+ TILs. A good prognosis was observed for patients with tumors that were highly infiltrated by CD20+ combined with CD4+ and CD8+, suggesting that CD20+ TILs support a CD4+ and CD8+ TIL-altered anti-tumor response. However, CD25+ and FOXP3+ Treg lymphocytes had no prognostic advantage based on Kaplan-Meier curves. Moreover, in multivariate analysis, CD20+ and FOXP3+ TILs had an opposite effect on RFS and OS. CD20+ TILs were correlated with a positive prognosis, and increased CD4+ and CD8+ TILs and FOXP3+ Treg lymphocytes. Therefore, our study suggested that TILs-B have dual and conflicting roles in TILs-T immune reactions in TNBC.

## Data Availability

The datasets used and/or analyzed during the current study available from the corresponding author on reasonable request.

## Abbreviations

TILs: Tumor-infiltrating lymphocytes
TILs-T: Tumor-infiltrating T lymphocytes
TNBC: Triple-negative breast cancer
TILs-B: Tumor-infiltrating B lymphocytes
Treg: T regulatory
FOXP3: Forkhead box protein 3
H&E: Hematoxylin and eosin
IHC: Immunohistochemistry
ER: Estrogen receptor
PgR: Progesterone receptor
HER2: Human epidermal growth factor receptor 2
ASCO/CAP: The American Society of Clinical Oncology/College of American Pathologists
FISH: Fluorescence in situ hybridization
CEP17: Chromosome enumeration probe 17
RFS: Relapse-free survival
OS: Overall survival
HRs: Hazard ratios
CI: Confidence interval
